# Iron diapirs entrain silicates to the core and initiate thermochemical plumes

**DOI:** 10.1038/s41467-017-02503-2

**Published:** 2018-01-04

**Authors:** J. R. Fleck, C. L. Rains, D. S. Weeraratne, C. T. Nguyen, D. M. Brand, S. M. Klein, J. M. McGehee, J. M. Rincon, C. Martinez, P. L. Olson

**Affiliations:** 1 Department of Geological Sciences, California State University, Northridge, Northridge, CA 91330 USA; 2 Department of Earth and Planetary Sciences, University of New Mexico, Albuquerque, NM 87131 USA

## Abstract

Segregation of the iron core from rocky silicates is a massive evolutionary event in planetary accretion, yet the process of metal segregation remains obscure, due to obstacles in simulating the extreme physical properties of liquid iron and silicates at finite length scales. We present new experimental results studying gravitational instability of an emulsified liquid gallium layer, initially at rest at the interface between two glucose solutions. Metal settling coats liquid metal drops with a film of low density material. The emulsified metal pond descends as a coherent Rayleigh−Taylor instability with a trailing fluid-filled conduit. Scaling to planetary interiors and high pressure mineral experiments indicates that molten silicates and volatiles are entrained toward the iron core and initiate buoyant thermochemical plumes that later oxidize and hydrate the upper mantle. Surface volcanism from thermochemical plumes releases oxygen and volatiles linking atmospheric growth to the Earth’s mantle and core processes.

## Abstract (web summary)

During planetary formation segregation of an iron core from rocky silicates takes place. Here, the authors use analogue fluid experiments show that iron diapirs entrain volatiles and silicates to the Earth’s core and initiate buoyant thermochemical plumes to reoxidize and hydrate the upper mantle and atmosphere.

## Introduction

Formation of iron cores and differentiation of terrestrial planets^1–3^ occurred rapidly, in only 10–30 My^3–5^, yet the style of iron descent to the core remains unknown. The process and path of iron descent has direct bearing on the chemical and thermal evolution of the mantle and core of all terrestrial planets. Planetary materials originally formed from dust and gas that condensed into particles within an accretionary disk^6^. Particles began to collide and clumped together forming kilometer-sized meteorites and small planetesimals. Differentiation began at this early stage by decay of radio nuclides, ^26^Al and ^60^Fe^7^. Heating from radioactive decay occurred at low pressure conditions within the mantles of small planetesimals. Planetesimals gradually increased in size (to hundreds of kilometers) where mantle temperatures would have reached ~2000 °K and ~20 GPA pressure^8^. Viscous dissipation from differentiation and descent of iron would have increased local mantle temperatures near the path of descent^2, 9^. As planetary embryos grew in size, silicate viscosities in the vicinity of impacts would have lowered due to localized impact heating and iron descent, but the bulk mantle viscosity would have been controlled by the peridotite solidus that is a strong function of pressure^10–12^. The combined effect of the decrease in the abundance of radio nuclides and the increase in bulk mantle pressures would have caused interior mantle temperatures to cool and viscosities to increase.

Impact collisions grew more infrequent but larger with time, becoming more violent causing partial melting of the impactor and target surface. The energetics and melting from moderate-sized impacts would have formed transient magma oceans or magma reservoirs^2, 5, 13^. The rheological base of the magma ocean is defined by melt fractions below 60%, at which depth, settling iron drops will pond^14^. We consider shallow (less than a few 100 km depth) magma oceans (see Discussion).

The mantles and cores of planetesimals previously differentiated by ^26^Al and ^60^Fe decay^7^ may have fragmented before merging with the proto-core of the planet^15, 16^. The late stages of bombardment may have involved a wide range of impact sizes. Fragmentation and breakup may be limited for giant impacts^17^, but these two processes are very likely to occur during impacts involving differentiated planetesimals and protoplanets. Once inside the magma ocean, turbulent mixing acts to shear liquid iron into smaller droplets^17–22^. The iron droplets will settle and accumulate in a metal pond at the base of the magma ocean^23^, at a depth where the silicate mantle is significantly more viscous^14^ due to growth of planetary radii and increasing mantle pressures.

While breakup of iron into drops during impacts seems probable, the nature of iron droplet descent to the core is not obvious. Iron descent to the core by percolation of liquid iron drops through a solid silicate matrix is a possibility, but is inhibited by large dihedral angles^1, 24^ and requires long, rather than short, core formation times. Metal percolation through solid silicate via interconnected networks is more likely in the lower mantle where shear stresses are higher^10, 25, 26^. The descent of liquid iron through fractures^27, 28^ has been proposed in the lower mantle if liquid metal−silicate viscosity contrasts are high. Large metal diapirs that form from Rayleigh−Taylor instabilities^1, 27^ are consistent with rapid core formation, but large metal diapirs would have residence times too short and surface area too small for wide spread metal−silicate equilibration in the upper mantle^29^. Here we consider the descent of metal diapirs consisting of emulsified liquid metal which simultaneously provides rapid core formation times and ample residence time for chemical equilibration at high pressures.

The main phase of accretion and iron descent in the Earth likely occurred in a reduced environment^30^. Trace element abundances of highly siderophile elements pervasive in the Earth’s upper mantle^31, 32^ indicate partial metal−silicate equilibration and suggest low oxygen fugacity prior to metal descent to the core^13, 33–36^. Atmospheric hydrogen was removed after the solar nebula dissipated^37^. Oxidation and hydration of the mantle is considered to be a later event, coming from several possible sources including hydrogen degassing or late stage arrival of highly hydrated and oxidized meteorites^6^. The “late veneer” model proposes that highly siderophile elements, volatiles, and oxides were added as the last few percent of Earth’s accretionary process^38^. An external model for this late veneer suggests migration of meteorites into outer asteroid belts beyond the snow line and later migration back towards the Earth^39^. An internal model suggests that disproportionation reactions involving perovskite produce Fe^+3^ in the lower mantle, which is then transported into the upper mantle by convection^33^. Late stage increase in mantle oxidation may also contribute to the late appearance of the oxygen atmosphere^37, 40^. Recent petrologic methods using vanadium as an oxybarometer^41^ indicates the rise in atmospheric oxygen is a gradual process leading up to the great oxygen event^42^ and may be linked to mantle convective processes or inner core growth.

Here we present the results of laboratory experiments using liquid metal gallium and glucose solutions to model the formation and gravitational instability of emulsified metal ponds in silicate magma oceans. Physical experiments using liquid metal have several advantages over numerical simulations^2, 43^, such as 3D geometry, multi-scale dynamics including turbulence, and the contrasting properties of surface tension, viscosity, and other rheological properties of liquid metal and viscous mantle silicates in planetary interiors. We show that emulsified metal ponds undergo Rayleigh−Taylor instabilities, forming metal-rich diapirs with trailing conduits. During planetary accretion, these metal-rich structures descend through the mantle entraining silicates and volatiles and deliver low density elements to the core. This mechanism provides an internally consistent model for rapid core formation, which first removes volatiles and oxides from the mantle, temporarily sequestering them in the core, and later returns them back to the upper mantle and atmosphere.

## Results

### Initial and experimental conditions

Our experiments model the evolution of an emulsified metal pond that settles within a magma ocean during or following a moderate-sized impact. The experiment is designed to characterize the boundary between a liquid metal layer overlying a viscous silicate mantle that is at or near the peridotite solidus^3, 14^ as described above. The underlying viscous mantle in our model assumes some degree of pre-differentiation due to melting from radioactive decay and metal−silicate segregation^6^. While a magma reservoir is expected to form from small- to moderate-sized impacts, our experiments are not intended to model the very largest, whole-planet melting impacts which involves core merging^15^. However, our experiments describe the state and growth of the target proto-core with entrained silicates that would be present before and after merging of a large differentiation event.

**Fig. 1:**
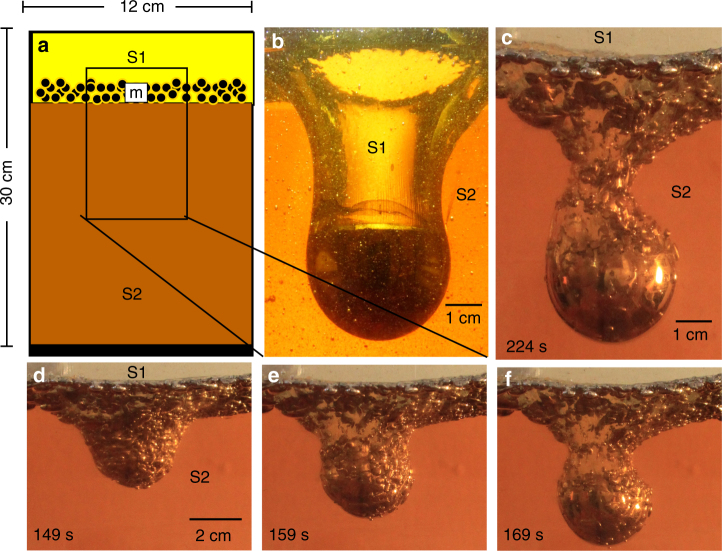
Gravitational instability of an emulsified metal pond. **a** Schematic of the initial conditions for each experiment (described in Methods). The metal emulsified layer (m) rests at the *S*1−*S*2 interface at *t*_*o*_. **b** Metal diapir and trailing conduit descending from a smooth metal pond. Photo image is cropped to the size of the small black box shown in **a**. All liquid metal collects quickly and descends in the first large diapir. Conduit material travels down and returns back up through conduit. **c** Metal diapir and trailing conduit descending from an emulsified metal pond (Experiment 14 at 224 s). Metal droplets descend in two stages. Conduit material travels down through the conduit and also by entrainment around each droplet. Time sequence of photographs (taken from a side view camera in spotlight) at **d** 149 s, **e** 159 s, and **f** 169 s showing the gravitational instability of an emulsified metal pond and formation of an emulsified diapir (Experiment 14). A later photograph of this experiment is shown in **c** at 224 s

A set of 34 laboratory experiments^44^, listed in Table 1, show gravitational instability of a pond consisting of emulsified liquid metal droplets (layer *m*) resting at the interface between a high viscosity fluid (*S*2) and low viscosity fluid (*S*1) as shown in Fig. 1. Non-dimensional parameters scaling our experiments to planetary interiors are described in Methods and Table 2. In each experimental case we vary a single parameter such as the viscosity of *S*1 or *S*2, thickness of the liquid metal pond (indirectly varying the radius of the metal diapir), or emulsion droplet size while holding all other properties fixed. Experiments test a wide range of densities for *ρ*_1_ and *ρ*_2_ (see Table 1). We choose *ρ*_1_ based on the range of densities estimated by petrologic studies of chondritic crustal melts^45^. Experiments presented here focus on the case of an emulsified liquid metal pond that forms by fragmentation and melting of small planetesimals on impact and turbulent mixing within a magma ocean (described above); however, there may be impact scenarios of moderate-sized meteorites with pre-formed cores that release partial volumes of liquid metal that are not significantly disturbed nor emulsified and which pool at the base of a magma ocean^1, 3, 27^. To consider this case, in four experiments (see Table 1) we also compare a coalesced smooth metal pond (Fig. 1b) to an emulsified metal pond (Fig. 1c).

**Table 1:** Laboratory fluid experiments

	*h*_m_	*μ*_2_	*ρ*_2_	*μ*_1_	*ρ*_1_	pond^a^	Variable
	cm	Pa ⋅ s	kg/m^3^	Pa ⋅ s	kg/m^3^	S/E	
1.	1.0	28,156	1520	1.06	1275	S	*μ*_2_
2.	1.0	21,274	1493	0.20	1298	S	*μ*_2_
3.	1.0	45,496	1510	0.07	1255	S	*μ*_2_
4.	1.0	8647	1444	0.06	1234	S	*μ*_2_
5.	0.5	27,480	1464	0.09	1270	E	*h*_m_
6.	1.0	27,480	1464	0.06	1254	E	*h*_m_
7.	0.38	27,480	1464	0.07	1244	E	*h*_m_
8.	0.25	27,480	1464	0.08	1256	E	*h*_m_
9.	0.38	27,480	1464	0.03	1260	E	*h*_m_
10.	0.25	7904	1491	0.10	1260	E	*μ*_2_
11.	0.25	36,698	1467	0.10	1262	E	*μ*_2_
12.	0.25	14,994	1476	0.08	1252	E	E/S^(b)^
13.	0.25	14,994	1476	0.09	1255	S	E/S^(b)^
14.	0.25	11,175	1417	0.10	1256	E	E/S^(b)^
15.	0.25	11,175	1417	0.09	1252	S	E/S^(b)^
16.	0.25	10,732	1460	0.02	1148	E	*μ*_1_
17.	0.25	10,732	1460	0.004	1125	E	*μ*_1_
18.	0.25	10,732	1460	0.90	1327	E	*μ*_1_
19.	0.25	10,732	1460	18.0	1373	E	*μ*_1_
20.	0.25	40,594	1463	0.10	1270	E	*r*_droplet_
21.	0.25	40,594	1463	0.10	1270	E	*r*_droplet_
22.	0.25	40,594	1463	0.07	1250	E	*r*_droplet_
23.	0.25	28,792	1514	0.13	1290	E	*μ*_2_
24.	0.25	42,737	1495	0.18	1282	E	*μ*_2_
25.	0.25	13,301	1419	0.17	1290	E	*μ*_2_
26.	0.75	18,376	1436	0.29	1296	E	*h*_m_
27.	0.25	5888	1459	1.65	1352	S	*μ*_1_
28.	0.25	5888	1459	12.65	1372	S	*μ*_1_
29.	0.25	5888	1459	0.001	970	S	*μ*_1_
30.	0.25	5888	1459	115.00	1385	S	*μ*_1_
31.	0.25	5888	1459	0.18	1296	S	*μ*_1_
32.	1.0	40,000	1460	–	–	S	*h*_m_
33.	0.25	40,000	1460	–	–	S	*h*_m_
34.	1.10	10,987	1513	0.01	1026	S	E/S

^a^Emulsified pond (E), Smooth pond (S)

^b^Compares an emulsified pond to a smooth pond

**Table 2:** Fluid properties and non-dimensional parameters

	Symbol	Units	Laboratory	Earth
Fluid 1 (S1)				
Viscosity	*μ*_1_	Pa ⋅ s	0.004–115	10^−3^–10^2^(^14^)
Density	*ρ*_1_	kg/m^3^	970–1385	2750–4000(^66^)
Surface tension	*γ*	mN/m	65	350(^53^)
Layer height	*h*_1_	m	0.025–0.045	10^5^–10^6^
Fluid 2 (S2)				
Viscosity	*μ*_2_	Pa ⋅ s	10^3^–10^4^	10^18^–10^21^
Density	*ρ*_2_	kg/m^3^	1417–1520	3300–5500
Layer height	*h*_2_	m	0.24–0.26	3×10^6^
Metal (M)				
Viscosity	*μ*_m_	Pa ⋅ s	10^−3^	10^−3^
Density	*ρ*_m_	kg/m^3^	5910	7000
Surface tension	*γ*	mN/m	707(^64^)	1720(^51, 52^)
Layer height	*h*_m_	m	10^−3^–10^−2^	10^2^–10^5^
Dimensionless parameters				
Reynolds # (droplets in S1)	*Re*_*S*1_	–	10^−2^–10^2^	10^−1^–10^7(a)^
Reynolds # (diapir in S2)	*Re*_*S*2_	–	10^−7^–10^−6^	10^−23^–10^−18(b)^
Reynolds # (*S*1_plume_ *in S2*)	*Re*_thch_	–	10^−11^–10^−8^	10^−21^–10^−19(c)^
Bond #	*B*_diapir_	–	8–50	10^14^–10^15^
	*B*_droplet_	–	10^−3^–10^−1^	10^−2^–10^0^
Buoyancy ratio	*ρ*_m_/*ρ*_2_	–	3.8–4.2	1.3–2.1
	*ρ*_2_/*ρ*_1_	–	1.1–1.6	1.4–2.3
Buoyancy diff	*ρ*_m_−*ρ*_2_	kg/m^3^	4390–4493	1500–3700
	*ρ*_2_−*ρ*_1_	kg/m^3^	150–550	1500–3100

Note: The range is given for the total difference, e.g. buoyancy *ρ*_m_−*ρ*_2_ (text written as (*ρ*_m−2_))

^a^Re determined using Stokes settling velocity and *r*_droplet_ = 10^−4^–10^−3^ m

^b^Re determined using Eq. (3) and *r*_diapir_ = 10^5^ m

^c^Re determined using Stokes velocity and *r*_thch_ = 10^4^–10^5^ m

### Descent of emulsified and smooth metal diapirs

**Fig. 2:**
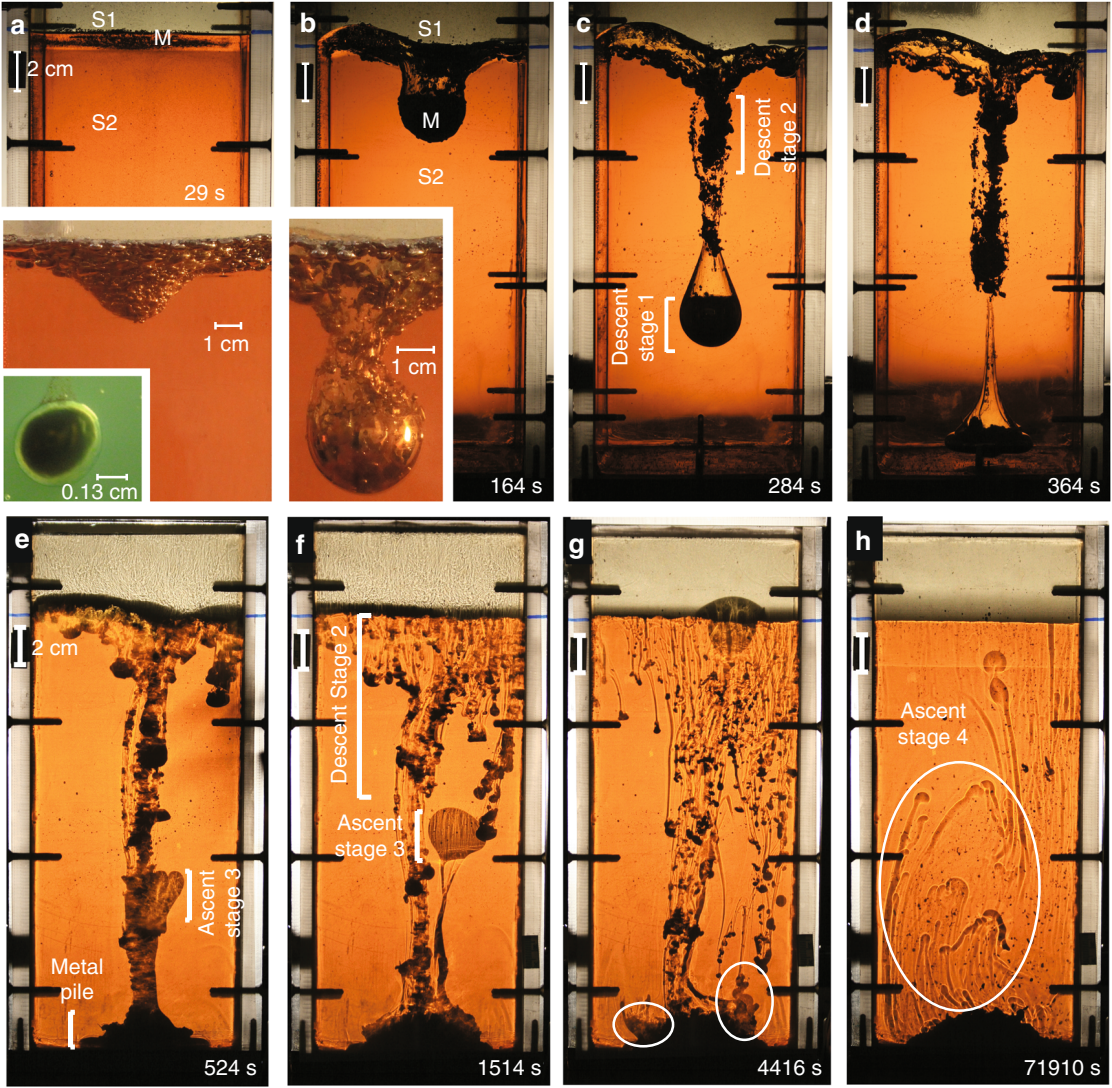
Descending metal diapir. **a** Starting conditions of a thin emulsified gallium layer resting at the interface between two glucose layers, *S*1−*S*2 (marked by a black tick mark on right side of tank). Upper inset: (orange light) A close up of the gallium (metal) layer, M, going unstable (photographed using a spotlight). Lower inset: (green light) Close up of the *S*1 film layer surrounding a metal droplet. **b** A diapir forms (stage 1) and a conduit develops. (Inset) Descent of stages 1 and 2 metal droplets. The droplets at the nose of the diapir coalesce due to shear stresses and form a smooth outer surface. It is unclear whether droplets within the diapir coalesce, due to the lack of visibility through the opaque metal. **c** A second stage of metal droplets descend behind the large diapir. Descent stage 2 is not observed in the case of a smooth metal pond (Fig. 1b). **d** Metal diapir reaches the base of the tank. Photographs **a**–**d** (Exp. 14 listed in Table 1) are taken in plain light. **e** A soliton forms at the base and is forced out of the conduit (shadowgraph). In the case of a smooth metal diapir (Fig. 1b) conduit material travels both downwards and upwards within the conduit. **f** The chemical plume (stage 3) is fully developed and travels vertically upward. Note the *S*1−*S*2 interface is now flat. **g** The chemical plume reaches the surface. At the base of the tank, the last, stage 4, of phase separation begins (white circles) between the *S*1 fluid and liquid metal pile. **h ***S*1 fluid segregates from the pile of metal droplets (20–30 h later) forming chemically buoyant plumelets. The diameter of each descending and rising plume head is governed by the density and viscosity ratios of the boundary layer and ambient fluid. Thus, the formation of the first large chemical plume is likely the maximum diameter for this viscosity ratio, but the small plumelets fall below this threshold. Fig. **e**–**h** (Exp. 14) are shadowgraphs

The major stages of formation and descent of a liquid metal diapir are shown in Figs. 1 and 2. Four stages of diapir descent and plume growth are observed in all emulsified experiments. We identify two stages of metal diapir descent and two stages of thermochemical plume ascent described here and in the next section. Emulsified liquid metal droplets (m) settle through a low viscosity and low density *S*1 fluid layer and are quickly coated with a film of low density *S*1 material (Fig. 2a inset) before coming to rest as a pond of coated metal droplets at the *S*1−*S*2 interface. (1) Stage 1 descent describes gravitational instability of the metal pond layer (Fig. 1d–f, Supplementary Fig. 1a) which generates a downwelling that collects quickly into a large nascent diapir of emulsified droplets (Figs. 1c and 2b). Metal droplets far from the initial downwelling site remain at the *S*1−*S*2 interface. The process of diapir formation in stage 1 is very similar to the smooth metal case (Fig. 1b); however in the smooth metal case, all of the ponded metal descends into the initial diapir.

### Formation of trailing conduits

**Fig. 3:**
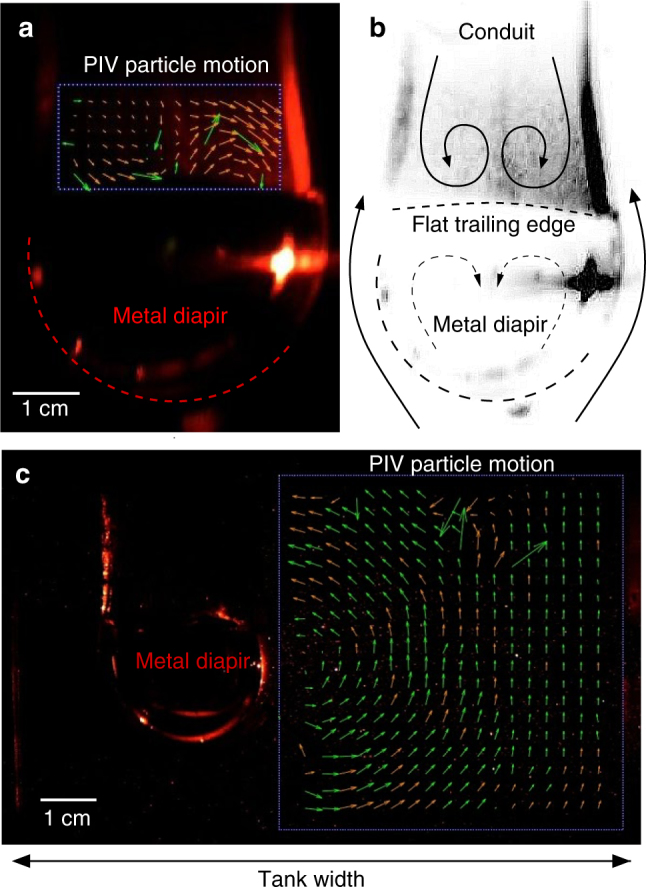
Flow visualization. **a** Particle image velocimetry (PIV) image capturing stream lines of circulation within a conduit. Image is dark due to photography taken in the absence of light illuminating the He-Ne laser sheet passing through the fluid experiment. Vectors indicate particle motion (silver-coated glass microspheres) suspended in *μ*_1_ during diapir descent and are tracked within the conduit only. Arrow length scales with fluid velocity. Green arrows indicate resolved particle motion between two frames taken at nine frames per second. Orange arrows are the interpolated vector field in time and space. (Note shorter vectors on left side of the conduit are caused by lower lighting not captured by the laser sheet and does not necessarily indicate slower fluid motion.) Dashed line is the inferred outline of the metal diapir. **b** Inverted negative image in gray scale of diapir and conduit descent as in **a**. Solid black streamlines indicate flow lines inside and outside the conduit which are resolved by the PIV vector field shown in **a**, **c**, respectively. Dashed streamlines and metal diapir outline are inferred. The PIV vectorization (green and orange arrows) indicates that drag along the inside of the conduit walls and at its base causes circulation at the base of the conduit which depresses the top of the descending metal diapir and flattens the top of the diapir surface shaping a liquid metal lower hemisphere during descent. **c** PIV image of a descending metal diapir (with particles seeded in *S*2 fluid only). PIV particle motion tracking is limited to right side of diapir only (taking advantage of symmetry). Return flow (green and orange particle motion vectors) surrounding the diapir extends to a lateral distance approximately equal to the diapir radius, beyond which edge effects are negligible. The flow field visualized on the right side of the diapir is used to infer the solid flow lines outside the diapir in **b**

Slow relaxation of the surrounding viscous *S*2 material causes *S*1 fluid to fill in the depression behind the sinking metal diapir, forming a conduit that is dragged downward (Fig. 2c, d). Circulation within the conduit walls and in the fluid surrounding the descending diapir is measured using particle tracking (see Fig. 3) and is shown to flatten the top of the metal diapir into a lower hemispherical shape (see Methods). (2) Stage 2 descent is observed as a second group of emulsified metal droplets slides down the evolving topography (Fig. 2c) at the *S*1−*S*2 interface towards the conduit. The delayed descent of droplets in stage 2 only occurs in emulsified metal pond experiments (Fig. 1c) and is not observed in smooth metal pond cases (Fig. 1b). This difference is due to the *S*1 film layer that coats each droplet in the former case. Droplets close to the initial downwelling site descend down steep topography at the *S*1−*S*2 interface created by the large initial downwelling. Coated droplets far from the initial downwelling site rest on approximately horizontal topography and maintain smaller masses that do not significantly depress the topography, lingering longer at the interface before descent. In the case of a smooth metal layer (Fig. 1b), the metal pond coalesces rapidly into a single large mass flowing quickly to form the initial downwelling (due to the extreme low viscosity and high density of liquid metal) with large negative buoyancy initiating descent in stage 1.

### Growth of thermochemical plumes

The conduit begins to collapse once the diapir reaches the base of the *S*2 fluid. Conduit fluid (*S*1) then reverses flow direction, separating from the metal droplets in the diapir and ascending through the conduit as a soliton^46^ as shown in Fig. 2e. (3) Stage 3 ascent is observed as the soliton rises (Supplementary Fig. 1b-d), meets the sinking metal droplets of descent stage 2 (Fig. 2e) and is forced out of the conduit (Fig. 2f) forming a chemically buoyant plume that rises to the surface along a new trajectory (consistent with Stokes velocity as shown in Supplementary Fig. 2). This exit of the chemically buoyant plume from the conduit is observed in all emulsion case experiments (see Supplementary Figs. 1 and 2). The influence of possible edge effects is determined using particle tracking methods and shown to be negligible (see Methods and Fig. 3c). After exiting the conduit, the chemically buoyant plume forms a spherical diapir with a thin tail, rising to the *S*1−*S*2 interface where it erupts into the *S*1 layer. The nature of the large plume head in stage 3 with a very thin tail results from the extreme viscosity ratios between the chemical plume and surrounding material^47^.

The second group of metal droplets continues its descent, adding to the pile of liquid metal at the base of *S*2 fluid (Fig. 2f). Topography of the basal metal layer is maintained in our experiments for >30 hours (Fig. 2h) due to the resistance of coated metal droplets to coalesce. (4) Stage 4 ascent is observed when the *S*1 film layer surrounding each metal droplet slowly segregates and migrates buoyantly out of the liquid metal pile forming late-stage plumelets (Fig. 2h). Some of the larger plumelets ascend along individual paths but most rise in merged trajectories, as packets or waves. Volume measurements of entrainment indicate that the bulk of the entrained *S*1 material is released slowly over time, but a very small volume is always retained in the liquid metal matrix.

### Experimental measurements and scaling

**Fig. 4:**
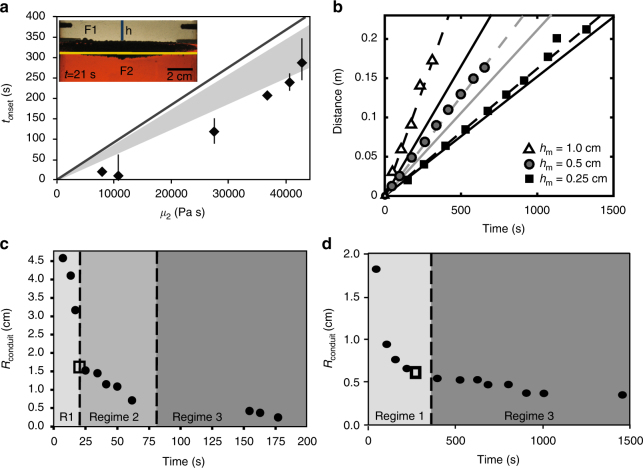
Diapir descent and conduit formation. **a** The measured onset time (two standard deviations) versus the viscosity of fluid S2 (black diamonds). The onset time is defined by the time when the horizontal metal pond layer begins to go unstable and sinks crossing the *S*1−*S*2 interface (yellow line, inset photograph). Theoretical prediction (black line) for onset of a Rayleigh−Taylor instability, Eq. (1) . Shaded region is revised theory using Eq. (5) for the range *h*_1_ from 2.5 to 4.2 cm. Experimental Data 8, 10, 11, 18, 21, and 24. **b** Descent distance versus time for a diapir during terminal velocity for three experiments which vary gallium layer thickness, *h*_m_ = 0.25 cm (black squares), *h*_m_ = 0.5 cm (gray circles), and *h*_m_ = 1.0 cm (open triangles). Classical Stokes theory for an inviscid sphere (solid line) considers a spherical radius using the actual volume of metal in the diapir. Revised Stokes theory considering reduced drag with a conduit including conduit mass from Eq. (2) is shown by dotted lines for each experiment (Experiments 5, 6, and 8). Average *r*_diapir_ is 0.017 m, 0.020 m, 0.025 m and *θ* is 20°, 30°, 55° for experiments 8, 5, and 6, respectively. The average conduit height, *h*_c_, is approximately half the descent distance. Errors in distance and time are smaller than the symbol. **c** Conduit radius versus time for a smooth metal pond with *μ*_1_ = 0.01 Pa ⋅ s and *μ*_2_ = 10,987 Pa ⋅ s (Experiment 34). Open square indicates the time when the metal diapir reaches the base of the tank. Three regimes for conduit behavior are identified (see text). **d** Conduit radius versus time for an emulsified metal pond (this study) with *μ*_1_ = 0.1 Pa ⋅ s and *μ*_2_ = 11,175 Pa ⋅ s (Experiment 14). Only regimes 1 and 3 are observed in all emulsified experiments

Linear stability theory for three layer fluids^48^ predicts our experimental system is gravitationally unstable; however, the onset time for the classical Rayleigh−Taylor instability^49^(solid lines) given by

1 $$t_{{\mathrm{onset}}} = \frac{{4\mu _2}}{{{\mathrm{\Delta }}\rho _{{\mathrm{m}} - {\mathrm{2}}}h_{\mathrm{m}}{\bf{g}}}}$$

under-predicts the onset times of the instabilities in our experiments (Fig. 4a). If Eq. (1) is modified to include the effect of all layers including the finite thickness of layer *S*1 (see Methods), an improved fit is obtained, as shown by the shaded area in Fig. 4a.

**Fig. 5:**
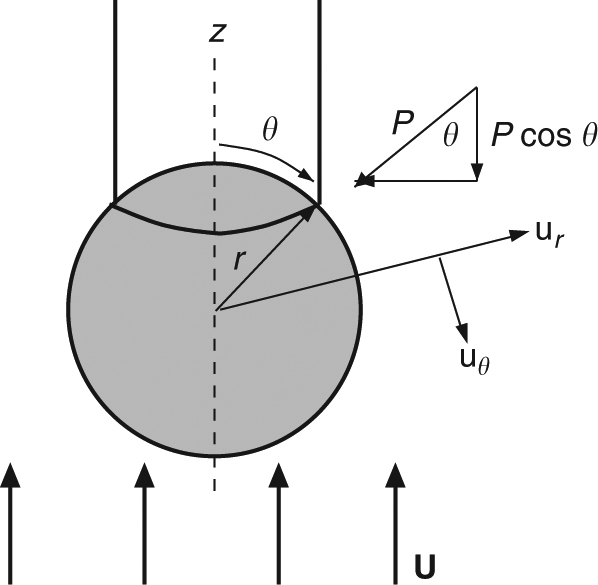
Geometry of a metal diapir and trailing conduit. Diapir of radius *r* and trailing cylindrical conduit are symmetric about the central axis (*Z*). The uniform flow field surrounding the sphere during descent is *U*. The angle *θ* is the polar angle between the *Z* axis and *r* as discussed in the text and Methods. Pressure is given by *P* and the components of velocity in the radial and polar (*θ*) direction is given by *u*_*r*_ and *u*_*θ*_.

The terminal velocity (**U**) of descending diapirs (Fig. 4b) in all experiments is observed to be faster than predicted by Stokes theory (solid lines). We add an additional term to the classical Stokes velocity (first term) which accounts for the reduced drag (second bracket) due to the presence of a conduit at the trailing side of the descending liquid metal sphere (see discussion and derivation in Methods).

2 $${\bf{U}} = \left({\frac{{{\mathrm{\Delta }}\rho _{{\mathrm{m}} - {\mathrm{2}}}r_{\mathrm{m}}^2{\bf{g}}}}{{3\mu _2}}} \right)\left({\frac{2}{{1 + \cos^3\theta }}} \right) - \frac{{{\mathrm{\Delta }}\rho _{2 - 1}n^2r_{\mathrm{c}}h_{\mathrm{c}}{\bf{g}}}}{{2\mu _2(1 + \cos^3\theta )}},$$

where *θ* is the angle between the vertical axis of the diapir and outer conduit wall and *n* = *r*_c_/*r*_m_ (see Fig. 5). Drag forces are maximum for shear stresses along stream lines which circulate around a perfect sphere during descent, but will be reduced if obstructed by the presence of a conduit on the trailing side of the sphere. The last term considers the mass of a trailing conduit (where *r*_c_ is conduit radius) and *h*_c_ is conduit height. The theoretical prediction given by Eq. (2) is shown in Fig. 4b (dashed lines). The departure of the diapir velocity from classical Stokes velocity (solid lines) increases systematically with *h*_m_ (which indirectly increases diapir radius, *r*_m_, once the metal pond goes unstable). Close fit with the theoretical prediction suggests that the increase in conduit radius (represented by *θ*), which forms behind each descending diapir, is the dominant factor controlling higher descent velocities.

### Conduit behavior

To study the formation of a conduit, we measure the change in average conduit radius versus time for the case of a smooth metal pond (Fig. 4c) and the case of an emulsified metal pond (Fig. 4d). Three distinct slopes indicate three regimes of conduit behavior for the case of a smooth metal diapir^50^ (Fig. 4c). In regime 1, conduits form as the metal diapirs descend. Conduit radius, governed by the leading metal diapir, is largest when the metal diapir first forms (Fig. 1b), and decreases in radius rapidly as it sinks to the base. Regime 2 marks conduit collapse due to the reversal of conduit material as *S*1 fluid rises up buoyantly through the conduit after the metal diapir reaches the base (open square) and stops sinking. The collapse rate is controlled by S1 buoyancy. The reduction in conduit radius is slower than during regime 1. In regime 3 thin conduits collapse slowly (displaying a shallow slope) after most of the S1 fluid has exited and includes slow diffusion with the surrounding S2 material. Thin conduits (regime 3) are observed to persist, however, for long times.

**Fig. 6:**
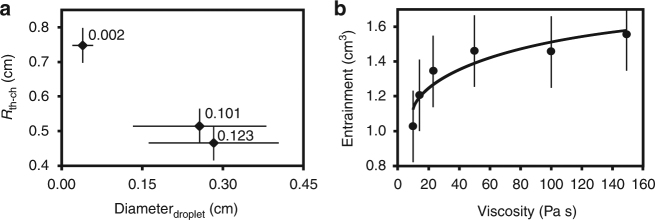
Entrainment by metal droplets. **a** The average radius of the chemical plume versus the average diameter of the emulsified droplets holding all other fluid properties constant. The Bond number values of each experiment are given at each point (Experiments 20–22). **b** Entrainment around a single solid metal sphere measured as a function of *μ*_1_ determined at a constant depth (5 cm) from the interface. A best fit curve (black line) indicates entrainment approaches an asymptotic limit above *μ*_1_ > 100 Pa ⋅ s. Entrainment around single liquid metal droplets is not yet measurable within error likely due to internal circulation, reduced drag, and imaging limitations. Our experiments using large volumes of liquid droplets (Fig. 2a–h) nonetheless entrain a significant volume of *S*1 fluid and is expected to depend at least moderately on *μ*_1_

**Fig. 7:**
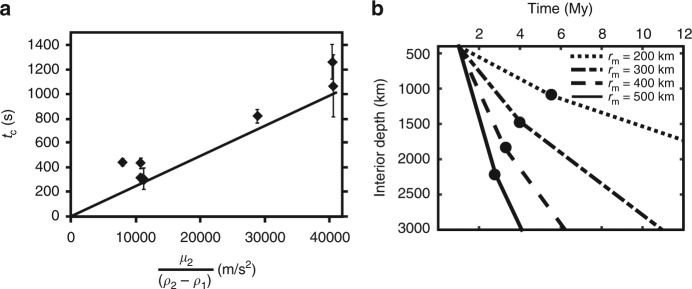
Conduit stability. **a** Conduit constriction time for seven experiments which vary *μ*_2_. Constriction time, *t*_c_ (see Results),  is defined by the time interval from the opening of a conduit when a diapir is fully formed (*R*_start_) to the time when the conduit radius stops changing after the metal diapir reaches the base of the tank base (Fig. 2d). Solid line shows theoretical prediction, *t*_c_, for a smooth metal diapir.  **b** Theoretical prediction for diapir descent time versus conduit constriction time^18^ (filled circles) for diapir radii (*r*_m_) 200–500 km using Eqs. (2) and (5) and previous theoretical analysis^18^. Each line represents the descent of a diapir in time and distance from the base of the magma ocean to the CMB after onset of the metal pond instability (assuming *ρ*_1_ = 2850 kg/m^3^, *ρ*_2_ = 4500 kg/m^3^, *μ*_2_ = 10^23^ Pa ⋅ s, average *h*_c_ = 300 km, and *θ* = 55°). Filled black circles indicate the time for conduit collapse for each diapir. An average onset time, *t*_onset_ of 1.0 My is used for a 1 km deep metal pond. Increasing *t*_onset_ will only prolong final diapir descent times but does not change the depth to which conduits stay open, as conduit constriction times are referenced to the beginning of conduit formation after *t*_onset_. Conduit radius, represented by *θ*, reduces drag around a diapir and is one of the most important factors controlling the depth of conduit closure showing that conduits stay open to deeper depths for increasing *θ* and higher *ρ*_1_. Constriction times, *t*_c_, are multiplied by 2, consistent with our laboratory observations (Fig. 7a). Once the conduit closes, diapirs are assumed to descend according to classical Stokes velocity (slope change). Interior mantle depth is defined from the base of a 400 km deep magma ocean. The total times estimated for metal plume descent and thermochemical plume ascent is 2–60 My for *μ*_2_ ranging from 10^19^ to 10^23^ Pa ⋅ s (see Supplementary Fig. 3), diapir radius of 500 km, and a soliton radius of 50 km

Conduit behavior for the case of an emulsified metal pond is shown in Fig. 4d. The steep slope identifying the rapid decrease in conduit radius due to rapid descent of the metal diapir is clearly observed in regime 1 and is similar to conduit behavior for the smooth diapir in Fig. 4c. The very shallow slope observed in regime 3 indicates very slow collapse of the conduit similar to regime 3 for a smooth metal diapir (Fig. 4c). However, the intermediate slope of regime 2 observed for a smooth metal diapir (Fig. 4c) is not observed for the case of an emulsified diapir in Fig. 4d. The absence of regime 2 indicates that the return flow of conduit material does not occur within the conduit. This is shown in Fig. 2e where buoyant material rises briefly in the conduit as a soliton and exits the conduit forming a chemically buoyant plume that rises to the surface along an independent pathway. We find that the amount of S1 fluid entrained into an emulsified metal diapir is controlled by the size of metal droplets (Fig. 6a) and S1 viscosity (*μ*_1_); larger thermochemical plumes result from smaller *r*_m_ and larger *μ*_1_ (Fig. 6b). The longevity of a conduit is described by the average constriction time and given in previous studies^43^ by $t_{\mathrm{c}} = \mu _2{\mathrm{/}}\left({{\mathrm{\Delta }}\rho _{2 - 1}gr_{\mathrm{c}}} \right)$. Constriction time of a conduit trailing behind an emulsified diapir depends on *μ*_2_ and Δ*ρ*, as shown in Fig. 7a, and is longer than theoretical predictions^18^ for a smooth metal pond (solid line) by a factor of ∼2, indicating internal circulation within the emulsified diapir is not efficient and retains S1 buoyant fluid.

## Discussion

Extrapolation of our experiments to the Earth’s mantle depends on how magma coats liquid iron droplets and their interfacial tension (IFT). We measure IFT in our experiments (see Methods), which causes S1 fluid to adhere to metal droplets and we obtain IFT = 642 mN/m. Liquid Fe ^51, 52^ at 1570–1823 °K and silicate magma^53^ gives ${\mathrm{IFT}}_{\gamma _{\mathrm{m}} - \gamma _1}$ ≈ |1720−350| = 1370 mN/m indicating that coating of iron droplets by molten silicate magma in the Earth is stronger than in our experiments. The resistance of magma-coated metal droplets to coalesce and the rapid growth of the pond instability (Figs. 1d and 4a) suggests that silicates in the magma film may remain chemically isolated from the magma ocean as the diapir forms during stage 1 descent. The metal pond instability time is the rate controlling process for metal descent. The time for gravitational instability of a metal pond to form a metal diapir is estimated using Eq. (5) (for *h*_m_ in the range 1–10 km and *μ*_2_ in the range 10^18^–10^21^ Pa ⋅ s) giving 0.045–45 Ky. Time for growth of this instability is significantly shorter than magma ocean cooling times estimated in recent numerical models^54^ and suggests that metal ponds go unstable and descend quickly, and may sequester O, H, other volatiles, or enriched elements within the film layer. These estimates use a lower bound for *h*_m_ and upper bound for *μ*_2_ emphasizing that growth of the instability is significantly shorter than magma ocean cooling times even with larger metal volumes or lower viscosities.

The entrainment volume is much smaller than the silicate/metal mass fraction of ~10/1 required for partial equilibration estimated from isotopic ratios and siderophile abundances^13^. We observe entrainment indicating estimated mass fractions that range from 1/5 to 2/5 indicating a finite silicate mass that limits the amount of metal−silicate chemical transfer^55^ in stage 1. In contrast, stage 2 metal droplets are delayed and more dispersed in the magma, allowing for more chemical transfer. The amount of siderophiles (including tungsten), per unit metal mass, extracted from silicates can be far smaller during stage 1 compared to stage 2 (see Supplementary Note 1) complicating the interpretation of core formation time^4^ in terms of Hf-W systematics. Small residual particles^19^ (<0.1 cm in Fig. 2g) remain at the *S*1−*S*2 interface; re-mixed would also contribute to upper mantle siderophile abundances^3, 33^. Partitioning of *O*, *S*, and other species between magma and metal droplets (inset Fig. 2a) within the descending diapir and during stage 2 metal descent (Fig. 2c) provides a source of light elements for the core.

The stability of a conduit during iron diapir descent is estimated by comparing the conduit constriction time, *t*_c_ (Fig. 7a) to metal pond instability time, *t*_onset_ (Methods), and diapir descent time (from Eq. (2)), depicted in Fig. 7b. Conduits stay open to depths ranging from 1000 to 2300 km depending on diapir radius, *r*_diapir_, and *θ*. This suggests that high pressure and temperature partition coefficients^33, 34^ for siderophile abundance and oxidation may apply to the equilibrium within a conduit, rather than an extraordinarily deep magma ocean^16, 56^. High pressures in the lower mantle will have the effect of increasing mantle viscosity, as shown in Fig. 7a, and to delay conduit closure behind descending metal diapirs.

We suggest that the silicate phase within a trailing conduit may be at least partially molten. While descent of large metal diapirs is shown to cause shear heating in the surrounding mantle^9, 36^, numerical studies of descending emulsified metal droplets indicate that heat is generated in both the metal and silicate by viscous dissipation^57^ and induces a ~1000 °K temperature increase in the metal phase that super heats the core. High pressure shock compression experiments^58^ indicate that these temperatures maintain silicates above the liquidus at core-mantle boundary (CMB) pressures (see Supplementary Note 2). Therefore silicates entrained within conduits of emulsified diapirs descending to CMB depths may exist as partial melt.

**Fig. 8:**
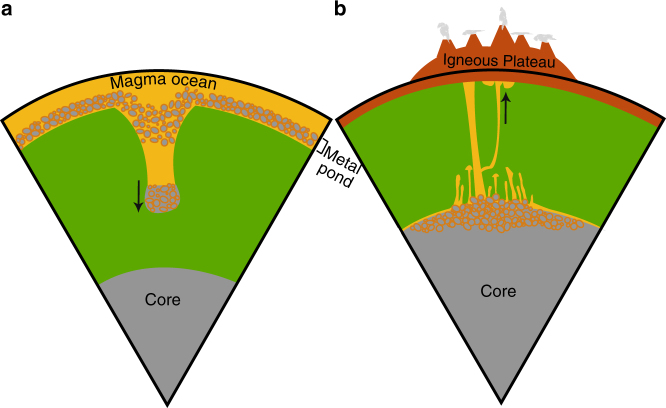
Interior schematic of iron diapir descent and rise of thermochemical plumes. **a** depicting the descent of a metal−silicate diapir (stage 1) from an emulsified metal pond, second-stage descent of remaining metal droplets (stage 2) and **b** ascent of a thermochemical plume (stage 3) and ascent of late stage plumelets (stage 4) from a metal pile at the base of the mantle

Extraction of silicate melt from metal piles formed at the CMB cools the core and may initiate and contribute to generation of the dynamo. Entrainment within the metal diapir delivers light elements to the core. The bulk density of the emulsified diapir dictates that it will descend below the CMB but the coated metal drops may distribute and reside at the top of the outer core creating temporary stratification due to buoyancy contrasts with heavier coalesced iron metal below (see Fig. 8b). Subsequent migration of the entrained silicate material out of the core maintains the dynamo and may be independent of giant impacts or inner core growth^59–61^. Prolonged residency of soluble light elements may dissolve in the outer core.

Our experiments show that the silicate melt entrained in the diapir segregates from the liquid iron phase in the conduit and at the CMB once the iron diapir enters the core. At this point, viscous heating ceases and the silicate melt cools by conduction. We compare conductive cooling times to advection where magma cooling times of 30–800 My (assuming thermal diffusivity, *κ* = 10^−6^ m^2^/s, and the radius of a thermochemical plume, *r*_thch_ > 50 km) are consistent or longer than the total time for diapir descent and thermochemical plume advection and ascent of 30–60 My (assuming *r*_m_ = 200–500 km, *r*_thch_ ~ 150 km). This suggests that upwelling thermochemical plumes may consist of at least partial melt. The density anomaly of a thermochemical plume, Δ*ρ* = *ρ*_2_−*ρ*_1_, ranges from 700 to 2000 kg/m^3^ and is significantly larger than a purely thermal mantle plume where Δ*ρ* typically varies from 10 to 100 kg/m^3^, implying that large chemically buoyant plumes rise rapidly, and are therefore little affected by solid-state thermal convection currents.

Arrival of a large thermochemical plume at the Earth’s surface would initiate volcanism and large igneous plateaus (Fig. 8). Oxygen and hydrous phases from the first large pulses of volcanism (ascent stage 3) are likely to escape the early weak atmosphere of the Earth^37^. The rise of plumelets in stage 4 (Fig. 8b) occurs over a longer duration ranging from 50 to 100 core formation times in our experiments. Scaling to the Earth suggests mantle plumelets (ascent stage 4, Fig. 2h) migrate out of the iron core matrix and rise slowly to the surface, consistent with Large Low Shear Velocity Provinces^62^ (see discussion of LLSPV structures in Supplementary Note 3), to oxidize and hydrate the upper mantle over 0.5 – 5.0 By. Out-gassing from volcanism of oxidized mantle elements may contribute to the rise of atmospheric oxygen.

Volcanic out-gassing from oxidized plumelet material would then contribute to the rise of atmospheric oxygen over many hundreds of millions of years. This is consistent with a gradual accumulation of atmosphere O_2_, leading up to the great oxygen event 2.3 Ga^37^ and is consistent with recent petrologic studies^41, 42^ that indicate mantle convective processes may contribute to atmospheric growth.

## Methods

### Initial conditions

The initial conditions in each experiment consist of three fluid layers, which model the Earth’s surface after a meteorite impact during accretion^63^. The experimental setup (Fig. 1a) consists of a top layer (*S*1) which represents the low viscosity silicate magma ocean and the lower layer (*S*2) which represents the viscous solid silicate mantle. A thin layer of emulsified liquid metal droplets (*m*) rests at the *S*1−*S*2 interface and represents liquid iron broken into droplets^14, 35^. All glucose solutions used for the two component fluid system are Newtonian. Fluid 1 (*S*1) consists of a diluted glucose solution. Fluid 2 (*S*2) is dehydrated by evaporation to produce highly viscous glucose solutions. Liquid gallium is immiscible with glucose solutions and demonstrates high density, low viscosity, and low melting temperature at ~30 °C making it useful for laboratory studies of planetary core dynamics. The buoyancy differences for our lab materials are compared to terrestrial planetary mantles in Table 2 and discussed below. Prior to each experiment, gallium is warmed and melted in a water bath at 30 °C and then mixed with *S*1 solution and agitated to form emulsion droplets. Small droplets broken up individually by release into glucose solutions through a syringe produces the same effect but is less time efficient. The surface tension of liquid gallium depends linearly on temperatures^64^ giving 707 dyne/cm at 30 °C (see Table 2). Surface tension for glucose solutions, measured using a contact angle analyzer at the University of California Los Angeles Fluid Mechanics Engineering laboratory, is mildly dependent on viscosity at 65 mN/m for *μ*_1_ = 0.04 Pa ⋅ s and 89 mN/m for *μ*_1_ = 156 Pa ⋅ s. The experimental tank has dimensions 12 cm×12 cm×30 cm with a copper base and four 1 cm thick Plexiglass sides. All experiments are conducted with an initial gallium layer thickness (*h*_m_) of 0.25 cm and an *S*2 layer depth of ~25 cm (except when testing the thickness of these layers).

Experiments are all conducted at constant temperature. Each experiment duration varies from several days to a few weeks and varies one physical parameter while holding all other properties fixed. The major features and stages of a liquid metal diapir descent shown in Fig. 2 are observed in all 21 experiments which consider an emulsified metal pond with only minor variations in conduit radius (*r*_c_), boundary layer growth rates, and descent/ascent velocities between experiments. Several types of visualization and lighting techniques are used. Plain light shines a light behind the experiment and illuminates the metal (descending) diapir best. Shadowgraphs resolve subtle density differences in the fluid (invisible to the naked eye) by light refraction and are used to visualize the small density contrasts between the conduit, thermochemical plumes, and S2 fluids.

### Non-dimensional parameters

Dimensionless parameters scale experiments to planetary interiors. The Reynolds number (Re), describes the ratio of inertial forces to viscous forces, and is defined as

3 $$\mathrm{Re} = \frac{{{\it{\rho }}{\bf{U}}L}}{\mu },$$

where *ρ* is the density of the droplet, diapir, or plume, **U** is the particle velocity, *L* is the length scale defined as *r*_droplet_, the radius of the droplet, diapir, or plume, and *μ* is the viscosity of the surrounding fluid. Small Re < 1 is governed by the Stokes flow regime where viscous forces dominate and inertial forces are negligible. For Re > 1, inertial forces dominate over viscous forces. The Reynolds number in the Earth silicate mantle (Re_*S*2_) is on the order of 10^−23^–10^−18^ for a metal diapir (see Table 2). Although the Re for metal diapirs in our experiments are larger, ranging from 2.9×10^−7^ to 1.9×10^−6^, the Reynolds number is small, Re << 1, in both systems indicating that viscous forces dominate and inertial forces are negligible providing appropriate scaling for planetary interiors. The Re_thch_ for rising chemical plumes in our experiments is between 5.5×10^−11^ and 4.6×10^−8^ compared to smaller values of 10^−21^−10^−17^ expected in planetary interiors. Both systems, however, are governed by the Stokes flow regime, Re << 1, where viscous forces dominate and inertial forces are unimportant. We also define a Reynolds number for descending droplets in the *S*1 layer where Re_*S*1_ ranges from 10^−2^ to 10^2^ in our experiments and spans the same range of values below and above the Stokes flow regime (see Table 2) that may be expected within a planetary magma ocean^14^.

The Bond number (*B*) determines the shape of the metal droplet or diapir and is defined by the ratio of buoyancy forces to surface tension. The Bond number is given by

4 $$B = \frac{{{\mathrm{\Delta }}\rho {\bf{g}}{\it{r}}^2}}{\gamma },$$

where Δ*ρ* is the density contrast between the droplet or diapir and surrounding *S*2 fluid, *g* is gravitational acceleration, *r* is the radius of the droplet or diapir, and *γ* is the surface tension of the metal droplet in *S*2. In the case *B* > 1, buoyancy forces overcome surface tension and the droplet or diapir will deform from a spherical shape. Descending diapirs in our experiments (Table 2) have *B*_diapir_ = 8–50 and although this is smaller than the range expected in planetary mantles of 10^2^–10^15^, Bond numbers are large, *B* > 1, in both systems indicating that buoyancy forces dominate over surface tension and metal diapirs will deform from a spherical shape in the Earth. This is exemplified by the hemispheric shape of diapirs observed in our experiments (Figs. 1 and 2). We find that conduit radius (represented by *θ*) increases for large *B* diapirs and reduces the drag around the diapir (see Supplement) traditionally assumed in the Stokes velocity case^65^. *B*_droplet_ defined for individual metal droplets is small, ranging from 10^−3^ to 10^−1^, in our experiments consistent with *B* expected for an emulsified metal pond^35^ within a magma ocean ranging from 10^−2^–10^0^ indicating that surface tension dominates and droplets remain spherical. The density difference (*ρ*_2_−*ρ*_1_) is lower in our experiments compared to the Earth’s interiors^45, 66^ (see Table 2). The density difference (*ρ*_m_−*ρ*_2_) is slightly higher in our experiments than observed for planetary interiors. The consistency of our data with theoretical predictions over a wide range of *μ*_2_ and Δ*ρ*, shown in Fig. 7a, suggests the ratio of these parameters in our results is applicable to planetary interiors. Assumptions for *ρ*_1_ used in Fig. 7b range from 2850 to 3700 kg/m^3^ obtained from previous studies^45, 66^ which estimate the density of chondritic silicate melts and the pressure dependence of silicate melt density in the deep Earth. This range of *ρ*_1_ does not affect the conduit closure depth significantly as *ρ*_1_ appears in both terms for diapir descent velocity (*U*) and constriction time^18^.

The onset time for the gravitational instability of the metal layer (Fig. 4a) is faster than predicted for a classic Rayleigh−Taylor instability. The classic Rayleigh−Taylor instability (shown by the solid line in Fig. 4a) is given in Eq. (1) from previous studies^49^. This classical treatment considers a single dense layer of thickness, *h*_m_, overlying a viscous medium. The fit is improved if we also consider the finite thickness of the *h*_1_ layer (*S*1), which overlies and descends with the *h*_m_ layer, leading to

5 $$t_{{\mathrm{onset}}} = \frac{{4\mu _2}}{{{\mathrm{\Delta }}\rho _{{\mathrm{m}} - 2}{\bf{g}}h_{\mathrm{m}} + \rho _1{\bf{g}}h_1}}.$$

The additional term in the denominator accounts for the mass of both the *h*_1_ and *h*_m_ layers as well as displacement of additional mass in the underlying *S*2 layer suggesting a multi-stratified layer participates in the instability as observed in our experiments. Equation (5) provides a better theoretical fit (shaded area in Fig. 4a) to the observed data compared to the classical prediction (solid line) which assumes a single layer of instability overlying a fluid half-space.

### Experimental measurements

We measure the dynamic IFT between two fluids by using the Drop Volume Method. Here we submerge a capillary tube filled with gallium below the surface of a container filled with *S*1 fluid. As the liquid metal is released, the drop grows at a rate modulated by the capillary wall separation, gravity, and the density difference with the surrounding fluid, (*γ*_IFT_ = *V*_drop_Δ*ρgπ*/*d*). The volume (*V*_drop_) is measured by the largest drop radius before it detaches from the tube. We measure IFT in our experiments, which causes *S*1 fluid to adhere to metal droplets, by the Drop Volume method giving ~500 mN/m. We predict the IFT for our fluids using the Antonoff Rule (the absolute difference in surface tension, *γ*, between each fluid) giving *γ*_m_−*γ*_1_ ≈ 642 mN/m, only a slightly larger IFT than our measurement. Antonoff’s Rule applied to planetary interiors with Fe liquid^51, 52^ at 1570–1823 °K and silicate magma^53^ gives ${\mathrm{IFT}}_{\gamma _{\mathrm{m}} - \gamma _1}$ ≈ |1720−350| = 1370 mN/m.

The average conduit radius (Fig. 4c, d) is measured in each photograph by dividing the full length of the conduit into 20 rectangular sub-sections. The width of each box gives the average diameter of that sub-section. The average conduit diameter for one time shot is obtained by taking the average width of the sub-sections. The constriction time of a conduit^44, 50^ shown in Fig. 7a is defined by the time interval from the opening of a conduit^67^ when a diapir is fully formed (defining *R*_start_) to the time when the conduit radius stops changing significantly^44, 67^ (Fig. 2d). Descent stage 2 is not observed in the case of a smooth metal pond^50^. In the case of a smooth metal diapir (Fig. 1b) all conduit material travels down in the initial diapir and returns upwards within the conduit after the diapir reaches the base. For an emulsified metal pond, ascent stage 3 begins with a soliton^46^ that forms at the base and is forced out of the conduit (Supplementary Fig. 1). Our experiments indicate that collision of ascent stage 3 and descent stage 2 is predicted for planetary interiors, as the second stage of droplets will always follow the conduit walls which is a lower energy path than vertical descent through the solid silicate mantle. Likewise, ascent stage 3 will initially ascend through the existing diapir-formed conduit (Fig. 2e and Supplementary Fig. 1) opposed to any other higher energy path through the solid lower mantle (Fig. 8). Core forming metal−silicate plume events may occur one or multiple times during planetary formation collecting within a metal pond until it becomes sufficiently unstable and forming a gravitational instability and descending as a diapir to the core.

We test for edge effects in our experimental tank using particle image velocimetry imaging techniques (Fig. 3), which shows return flow around the diapir extends a distance approximately equal to the diapir radius. The width of the box is more than two times this distance in all experiments indicating that edge effects from our tank are negligible.

We do not directly account for pressure effects in our experiments. The extreme physical properties of liquid metal and silicate, however, are difficult to model directly in numerical simulations^16, 43, 68^ at length scales which consider break up or emulsification of liquid iron. High pressures in the lower mantle will have the strongest effect of increasing mantle viscosity (The magnitude of the increase in density is subtle by comparison). We show in Fig. 4a that increasing the ambient layer viscosity, *S*2 (or mantle viscosity) will slow the relaxation time to close the fluid-filled conduit. Numerical simulations^36^ as well as theoretical work studying Rayleigh−Taylor instabilities^27^ indicate that viscosities of 10^23^ Pa ⋅ s are not sufficient to prevent large metal diapirs (with a buoyancy difference *ρ*_m_−*ρ*_2_ ≥ 2000 kg/m^3^) from descending to the core. (The range of buoyancy differences in our experiments are consistent with lower mantle values, see Table 1.) Mantle viscosities in the early Earth are suggested to be even lower (10^18−21^ Pa ⋅ s) during accretion^14^ and would have the effect of increasing iron diapir descent velocities (compare Fig. 7b for *μ* = 10^23^ to Supplementary Fig. 3 for *μ* = 10^21^). Increased mantle viscosities in the lower mantle will respond slower to a magma-filled conduit that forms behind a large descending metal diapir delaying collapse of the conduit due to slowed viscous relaxation. The depth to which we can expect conduits to survive ranges from 1000 to 2300 km (see Fig. 7b) depending on diapir radius. Numerical studies^57^ of liquid iron descent through the mantle show that viscous dissipation causes heating of iron and silicates reaching temperatures sufficient to maintain silicate melt during descent through the mantle adiabat and at CMB pressures. The liquidus for mantle peridotite at lower mantle and CMB pressures is obtained by high pressure mineral studies^58^ using shock compression techniques and indicates the increased temperatures due to viscous dissipation lie above the liquidus during descent to the core (see discussion). Cooling times for molten silicates at the CMB may be protracted by as much as 3 By (see Supplementary Note 2) if thermal diffusivity is temperature-dependent^69^. High pressures are also shown to increase dissolution of Si and O in liquid Fe^70^.

### Reduced drag due to the presence of a conduit

We account for the reduced drag around a descending metal diapir due to the presence of a trailing conduit^67^. We first consider pressure drag (*D*_p_) and begin by assuming the contact point of the conduit wall with the sphere makes an angle, *θ*, with the vertical axis of the spherical metal drop (see Fig. 5). The component of the pressure along the direction of movement of the sphere is given by pcos*θ*. We use a ring surface element in spherical coordinates taking advantage of symmetry about the radius (*a* = *r*_diapir_). This simplifies to a single integral

6 $${{D}}_{\mathrm{p}} = {\int}_\theta ^\pi (p\cos\theta )2\pi a^2\sin\theta {\rm d}\theta ,$$

where *θ* is kept in the integral limit because the drag forces acting on the diapir will depend on the conduit radius *θ*. The pressure at *r* = *a* for an inviscid sphere^65^ depends on viscosity (*μ*_2_),

7 $$p = \frac{{\mu _2{\bf{U}}}}{a}\cos\theta ,$$

where *U* is the uniform flow field surrounding the sphere during descent. Solving for *p*cos*θ* and substituting into (6) yields

8 $${{D}}_{\mathrm{p}} = 2\pi a^2{\int}_{\theta} ^{\pi} \left({\frac{{\mu _2{\bf{U}}}}{a}\cos^2\theta } \right)\sin\theta {\rm d}\theta .$$

Integrating gives an expression for *D*_p_ in terms of *θ*

9 $${{D}}_{\mathrm{p}} = \frac{{2\pi a\mu _2{\bf{U}}}}{3}(1 + \cos^3\theta ).$$

Viscous drag (*D*_v_) can be expressed mathematically as the sum of the viscous stresses (*τ*) along the surface of the sphere. For an inviscid sphere, the component of viscous stress in the *θ* direction (Fig. 5) is zero. Thus the viscous drag is the sum of the components of viscous stress in the radial (*r*) direction

10 $${{D}}_{\mathrm{v}} = {\oiint} {\left({\tau _{rr}\cos\theta } \right){\rm d}A} .$$

Radial stresses (*τ*_*rr*_) depend on the change in velocity, *u*_r_ in the radial direction

11 $$\tau _{rr} = 2\mu _2\frac{{\delta {{u}}_{\mathrm{r}}}}{{\delta r}}.$$

For an inviscid sphere which has boundary conditions at *r* = *a* where radial velocities, *u*_*r*_ = 0, tangential stresses, *τ*_*rθ*_ = 0 vanish, it can be shown that

12 $${{u}}_r = {\bf{U}}\left({ - 1 + \frac{a}{r}} \right)\cos\theta .$$

Substituting (11) and (12) into (10) and simplifying gives

13 $${{D}}_{\mathrm{v}} = {\int}_{\theta} ^{\pi} \frac{{ - 2\mu _2{\bf{U}}\cos^2\theta }}{a}(2\pi a^2\sin\theta {\rm d}\theta ).$$

Integrating with respect to *θ* gives

14 $${{D}}_{\mathrm{v}} = \frac{{ - 4\pi a\mu _2{\bf{U}}}}{3}(1 + \cos^3\theta ).$$

Pressure drag (9) opposes the direction of viscous drag (14) and can be combined giving an expression for the total drag on the diapir,

15 $${{D}}_{{\mathrm{diapir}}} = {{D}}_{\mathrm{p}} - {{D}}_{\mathrm{v}} = 2\pi a\mu _2{\bf{U}}(1 + \cos^3\theta ).$$

Below we consider the sum of forces acting on the diapir including the mass of the diapir, mass of the conduit *c*, the buoyancy of the diapir with respect to *S*2, the buoyancy of the conduit with respect to *S*2, and the drag forces derived above

16 $$\mathrm{Mass}_{\mathrm{m}} + \mathrm{Mass}_{\mathrm{c}} = \mathrm{MassBuoyancy}_{m,2} + \mathrm{MassBuoyancy}_{c,2} + {{D}}_{{\mathrm{diapir}}},$$

shown as

17 $$\rho _{\mathrm{m}}{\bf{g}}V_{\mathrm{m}} + \rho _1{\bf{g}}V_{\mathrm{c}} = \rho _2{\bf{g}}V_{m,2} + \rho _2{\bf{g}}V_{c,2} + 2\pi r_{\mathrm{m}}\mu _2{\bf{U}}(1 + \cos^3\theta ).$$

Solving for velocity, **U**, gives

18 $${\bf{U}} = \frac{{\rho _{\mathrm{m}}{\bf{g}}V_{\mathrm{m}} + \rho _1{\bf{g}}V_{\mathrm{c}} - \rho _2{\bf{g}}V_{\mathrm{m}} - \rho _2{\bf{g}}V_{\mathrm{c}}}}{{2\pi r_{\mathrm{m}}\mu _2(1 + \cos^3\theta )}},$$

where *r*_m_ is metal diapir radius and *V* is volume. Expanding and combining like terms gives

19 $${\bf{U}} = \frac{{{\mathrm{\Delta }}\rho _{{\mathrm{m}} - 2}{\bf{g}}\frac{4}{3}r_{\mathrm{m}}^3 - {\mathrm{\Delta }}\rho _{2 - 1}{\bf{g}}r_{\mathrm{c}}^2h_{\mathrm{c}}}}{{2r_{\mathrm{m}}\mu _2(1 + \cos^3\theta )}},$$

where *r*_c_ is conduit radius and *h*_c_ is conduit height. If the conduit radius is written as a fraction (*n*) of diapir radius, *r*_c_ = *nr*_m_, this reduces to

20 $${\bf{U}} = \frac{{{\mathrm{\Delta }}\rho _{{\mathrm{m}} - 2}{\bf{g}}\frac{4}{3}r_{\mathrm{m}}^3 - {\mathrm{\Delta }}\rho _{2 - 1}{\bf{g}}\left({nr_{\mathrm{m}}} \right)^2h_{\mathrm{c}}}}{{2r_{\mathrm{m}}\mu _2(1 + \cos^3\theta )}}.$$

This can be simplified and written in terms of the Stokes classical velocity as

21 $${\bf{U}} = \left({\frac{{{\mathrm{\Delta }}\rho _{{\mathrm{m}} - 2}r_{\mathrm{m}}^2{\bf{g}}}}{{3\mu _2}}} \right)\left({\frac{2}{{1 + \cos^3\theta }}} \right) - \frac{{{\mathrm{\Delta }}\rho _{2 - 1}n^2r_{\mathrm{m}}h_{\mathrm{c}}{\bf{g}}}}{{2\mu _2(1 + \cos^3\theta )}}.$$

The first term is the classical expression of Stokes velocity for an inviscid sphere, the second bracket describes reduced drag due to the presence of a conduit, and the last term represents the mass of the conduit and displacement of *S*2 fluid.

Our results indicate that conduit radius, represented by *θ*, is the biggest factor influencing diapir descent velocities. Diapirs with larger Bond number (see Methods above) cause larger diapir shape distortion from a sphere displaying flatter, wider trailing edges, entrain wider conduits and have larger *θ* as shown in Fig. 4b. We set an average value for the factor *n* = 0.3 in Fig. 4b. If mantle viscosities were smaller (e.g. *μ*_2_~10^22^ Pa ⋅ s), diapirs descend faster (in less than 2 Ma, see Supplementary Fig. 3), increasing core formation times, but the relaxation time of *μ*_2_ to close conduits also increases and they stay open to about the same depth as shown in Fig. 7b.

Coefficients due to form drag (or shape change) of the metal diapir are not yet considered, but this would only reduce the predicted velocity. We show that diapirs descend much faster than the Stokes velocity prediction, indicating that reduced drag due to the presence of a conduit, represented in Eq. (2), is the dominant factor governing our experimental observations for increased diapir velocities.

### Data availability

All relevant data are available from the authors.

## Electronic supplementary material

Supplementary Information
