# Supplementary material for: Iron diapirs entrain silicates to the core and initiate thermochemical plumes
Source: Nat Commun. 2018 Jan 4;9:71. doi: 10.1038/s41467-017-02503-2 (PMC5754369; doi:10.1038/s41467-017-02503-2)
Supplement: Supplementary file 1 — Supplementary Information [file 41467_2017_2503_MOESM1_ESM.pdf]

Supplementary Note 1

Metal-silicate equilibration in an emulsified iron diapir. The volatile and oxygen concentration within the film layer surrounding each droplet (Supplementary Figure 1a) may also act to buffer or slow siderophile exchange between the surrounding magma ocean and the metal droplet and increase retention of siderophiles in the magma ocean. The metal diapir descending in stage 1 develops a trailing conduit which entrains a pocket of S1 material behind it that has minimal exchange with metal droplets (Fig. 2b-d), later contributing to the bulk volume of the upwelling thermochemical plume in stage 3. Numerical studies of shear heating from viscous dissipation of liquid iron descent[57] indicate that silicate magma, entrained within the conduits depicted here, is maintained as a melt and may be minimally fractionated during transport to the core. This indicates that upwelling thermochemical plumes (Fig. 2f, Supplementary Figure 1b-d and 2) following core formation events (stage 3) may deliver trace siderophiles as well as volatiles to oxidize and hydrate the upper mantle. Return flow via thermochemical plumes is consistent with geochemical observations for excess siderophiles as well as relative siderophile abundances[30], if rapidly upwelling plumes are not significantly fractionated during surface melting. Thermochemical plumelets (stage 4) are formed by migration and segregation of the film layer from droplets in the metal pile which is a mixture of droplets from stage 1 and stage 2 descent (Supplementary Figure 1a). Continued oxidation and hydration of the upper mantle proceeds slowly through rise of plumelets (Fig 2h) over time.

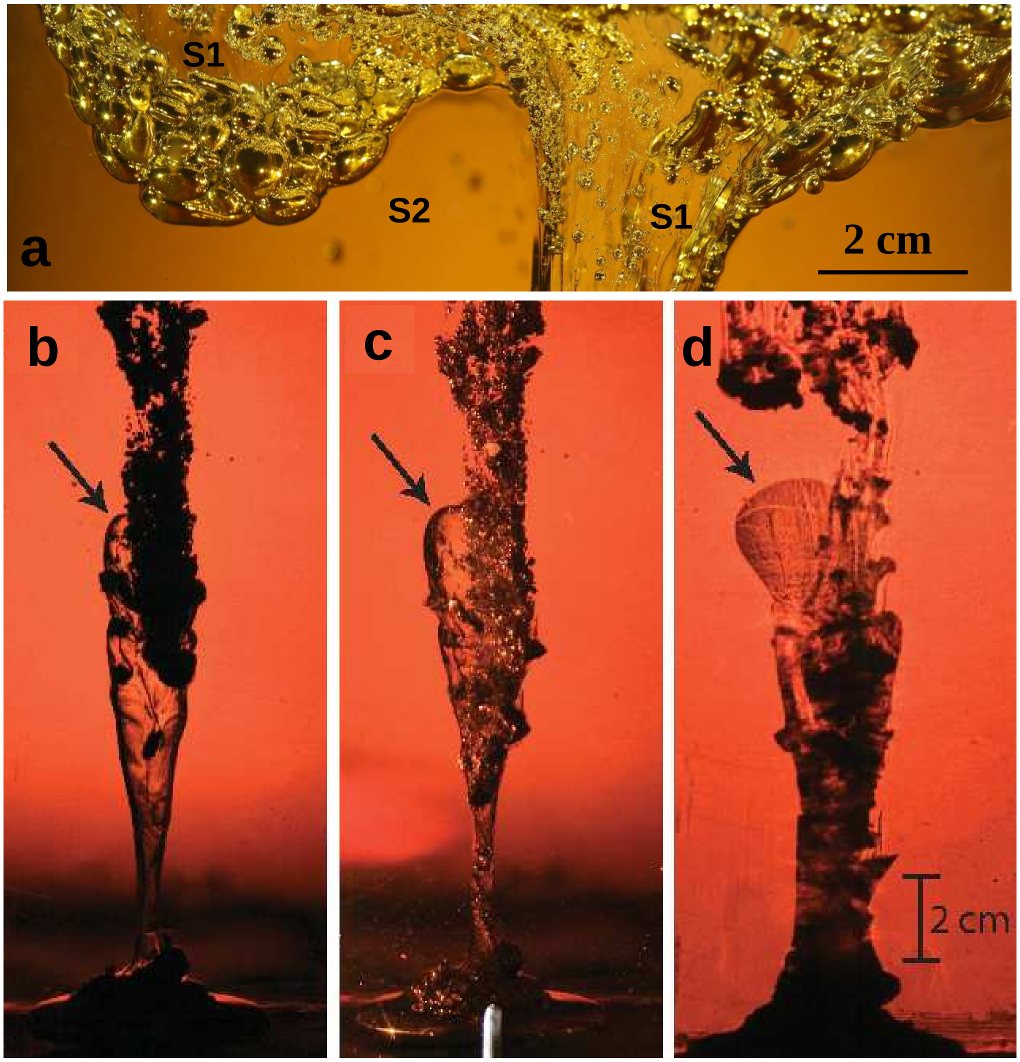

Supplementary Figure 1: Growth of conduits and solitons (a.) Photograph showing the development of an emulsified liquid metal diapir and trailing fluid filled conduit (S1) descending through the viscous (S2) layer. The first emulsified diapir (stage 1) descended near the center and only shows the trailing conduit (center of photo). A second emulsified diapir (left) is visibly forming. The film layer coating defines and separates each liquid metal droplet in the developing diapir and is clearly visible around several droplets. Delayed metal droplets far from the original downwelling slide down along the walls of the conduit (right) demonstrating stage 2 descent. (similar to Exp. 31 with $\mu_2 = 1100$ Pa·s). (b.) Close up photograph of a soliton developing at the base of a conduit after metal diapir descent with back lighting (c.) spot light and (d.) in shadowgraph (Exp. 10).

Supplementary Note 2

Silicate melt at the core-mantle boundary. Although silicate melt is compressible and is expected to increase in density with pressure and mantle depth[66], numerical studies show that shear heating from viscous dissipation of sinking iron diapirs[57] is sufficient to increase silicate temperatures above the liquidus reported in high pressure, temperature experiments of peridotite melts[58] at CMB conditions. Silicate melt present in the lower mantle at CMB pressures and temperatures may take a long time to cool. Silicate melt cooling times will be even longer if melt at the CMB is in contact with superheated liquid iron. Cooling times will also be protracted if thermal diffusivity is temperature-dependent[69] and is lowered ($\leq 10^{-7}$ m^2/s) by elevated silicate temperatures requiring as long as 3 By for conductive cooling.

Supplementary Note 3

Lingering conduits. This work and previous studies show that thin conduits linger after conduits empty, are resistant to complete collapse (Supplementary Figure 3) due to the increase in bulk density of conduit material[18,50] and may guide the pathways of the first thermally convecting mantle plumes that rise later. In our experiments, thin conduits last for long times (from a few days to 2 weeks). Scaling diapir descent and conduit collapse times to planetary interiors indicates that thin conduits in the Earth may continue to drain buoyant silicates slowly by migration out of metal piles at the core-mantle boundary for 0.5 - 5 By. Circulation from fully developed mantle convection is likely to interact with these lingering conduits, reducing their lifespan or intermixing the conduits and their contents with convective structures.

Our experiments indicate that while the bulk of the $S1$ material entrained into the metal pile is later released, a small percentage of the $S1$ film layer remains permanently intermixed within the liquid metal matrix. This suggests the lingering presence of small concentrations of silicates and volatiles in the iron core matrix may also contribute to the light element budget of the core, particularly Mg and Si[3,61]. Long lived metal piles and lingering metal-silicate residue suggests interaction across the CMB to release silicates from the core[70] and produce a high density[66] and low velocity basal layer at the CMB that can act to guide and stabilize the roots of later convecting mantle plumes[62]. Delivery of silicates and volatiles to the core and later release and ascent (Fig. 2h) of plumelets (ascent stage 4) from the CMB in (laterally stratified) groups or waves may be consistent with the timing and ancient signature of broad LLSVP (large low shear velocity province) structures observed in the lower mantle by global seismic tomography studies[62]. Mixing of entrained material within the core over time would distribute buoyant upwellings at regular wavelengths, $\lambda = 4\pi\delta[\mu_1/(180\mu_2)]^{1/5}$ predicted by Rayleigh-Taylor instabilities[49], suggesting the entrained material viscosity μ_2 is in the range 10^{18} $Pa \cdot s$ (assuming a wavelength of ∼11,000 km between LLSVP's, boundary layer thickness δ = 250 km, and μ_1 = 10^{23}).

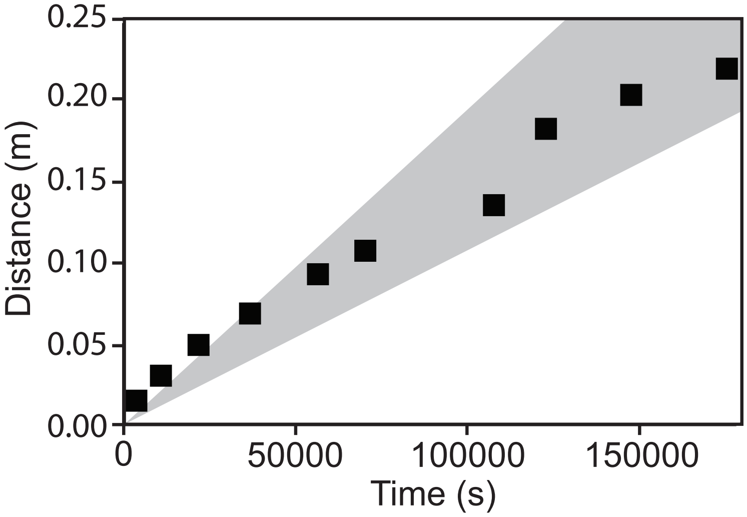

Supplementary Figure 2: Scaling of thermochemical plume ascent velocity with Stokes theory. The ascent distance of a chemical plume (stage 3) versus time (Exp 8). The Gray shaded region is the Stokes prediction between an inviscid sphere (coefficient of 1/3) and a rigid sphere (coefficient of 2/9) assuming the plume is made of $S1$ fluid. The plume velocity is consistent with the classical Stokes velocity prediction confirming its composition of $S1$ fluid and indicates that the plume is rising faster than diffusion time.

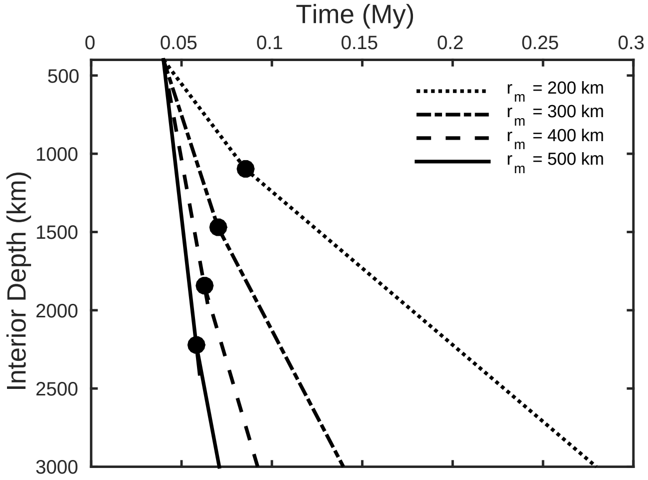

Supplementary Figure 3: Conduit stability. Diapir descent time versus conduit constriction time (filled circles) as in Figure 7b with $\mu_2 = 10^{21}$ and an average $t_{onset} = 0.04$ My. The collapse depth of the conduits are not changed significantly but descent times of a metal-silicate emulsified diapir in a lower viscosity mantle are shorter.
